# *N*,*N*′-Bis(4-chloro­phenyl­sulfon­yl)suberamide

**DOI:** 10.1107/S1600536811028662

**Published:** 2011-07-23

**Authors:** Vinola Z. Rodrigues, Sabine Foro, B. Thimme Gowda

**Affiliations:** a Department of Chemistry, Mangalore University, Mangalagangotri 574199, Mangalore, India; b Institute of Materials Science, Darmstadt University of Technology, Petersenstrasse 23, D-64287, Darmstadt, Germany

## Abstract

The asymmetric unit of the title compound, C_20_H_22_Cl_2_N_2_O_6_S_2_, contains one half-mol­ecule with a center of symmetry at the mid-point of the central C—C bond. The conformations of all the N—H, C=O and C—H bonds in the central amide and aliphatic segments are *anti* to their adjacent bonds. The mol­ecule is bent at the S atom with a C—SO_2_—NH—C(O) torsion angle of −80.6 (4)°. The dihedral angle between the benzene ring and the SO_2_—NH—C(O)—CH_2_—CH_2_—CH_2_ segment is 79.5 (2)°. In the crystal, inter­molecular N—H⋯O(C) and N—H⋯O(S) hydrogen bonds link the mol­ecules into chains along the *b* axis.

## Related literature

For our studies on the effects of substituents on the structures of *N*-(ar­yl)-amides, see: Gowda *et al.* (2000 ▶, 2007 ▶), on *N*-(aryl­sulfon­yl)-amides, see: Rodrigues *et al.* (2011**a* ▶,b* ▶) and on *N*-(ar­yl)-aryl­sulfonamides, see: Gowda *et al.* (2005 ▶).
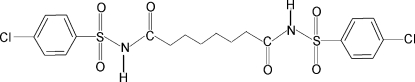


## Experimental

### 

#### Crystal data

C_20_H_22_Cl_2_N_2_O_6_S_2_
*M* *_r_* = 521.42
Monoclinic, 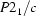

*a* = 21.925 (4) Å
*b* = 5.5855 (8) Å
*c* = 9.381 (1) Å
β = 93.91 (1)°
*V* = 1146.1 (3) Å^3^
*Z* = 2
Mo *K*α radiation
μ = 0.51 mm^−1^
*T* = 293 K
0.48 × 0.14 × 0.06 mm

#### Data collection

Oxford Diffraction Xcalibur diffractometer with Sapphire CCD detector
Absorption correction: multi-scan (*CrysAlis RED*; Oxford Diffraction, 2009 ▶) *T*_min_ = 0.793, *T*_max_ = 0.970
3854 measured reflections
2081 independent reflections
1522 reflections with *I* > 2σ(*I*)
*R*_int_ = 0.029

#### Refinement

*R*[*F* ^2^ > 2σ(*F* ^2^)] = 0.063
*wR*(*F* ^2^) = 0.115
*S* = 1.18
2081 reflections
148 parameters
1 restraint
H atoms treated by a mixture of independent and constrained refinement
Δρ_max_ = 0.29 e Å^−3^
Δρ_min_ = −0.32 e Å^−3^

### 

Data collection: *CrysAlis CCD* (Oxford Diffraction, 2009 ▶); cell refinement: *CrysAlis RED* (Oxford Diffraction, 2009 ▶); data reduction: *CrysAlis RED*; program(s) used to solve structure: *SHELXS97* (Sheldrick, 2008 ▶); program(s) used to refine structure: *SHELXL97* (Sheldrick, 2008 ▶); molecular graphics: *PLATON* (Spek, 2009 ▶); software used to prepare material for publication: *SHELXL97*.

## Supplementary Material

Crystal structure: contains datablock(s) I, global. DOI: 10.1107/S1600536811028662/sj6195sup1.cif

Structure factors: contains datablock(s) I. DOI: 10.1107/S1600536811028662/sj6195Isup2.hkl

Supplementary material file. DOI: 10.1107/S1600536811028662/sj6195Isup3.cml

Additional supplementary materials:  crystallographic information; 3D view; checkCIF report

## Figures and Tables

**Table 1:** Hydrogen-bond geometry (Å, °)

*D*—H⋯*A*	*D*—H	H⋯*A*	*D*⋯*A*	*D*—H⋯*A*
N1—H1*N*⋯O2^i^	0.85 (2)	2.19 (3)	2.975 (4)	153 (4)
N1—H1*N*⋯O3^i^	0.85 (2)	2.57 (3)	3.227 (4)	135 (3)

Symmetry code: (i) 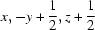.

**Fig. 1.**
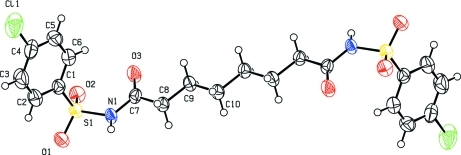
Molecular structure of (I) showing the atom labelling scheme. Displacement ellipsoids are drawn at the 50% probability level and H atoms are represented as small spheres of arbitrary radii.

**Fig. 2.**
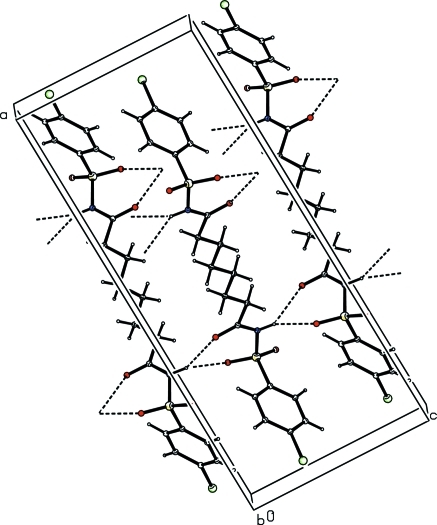
Molecular packing of the title compound with hydrogen bonding shown as dashed lines.

## supplementary crystallographic information

### Comment

The amide moiety is an important constituent of many biologically significant compounds. As part of our studies on the effects of ring and side chain substitutions on the structures of *N*-(aryl)-amides (Gowda *et al.*, 2000, 2007), *N*-(arylsulfonyl)-amides (Rodrigues *et al.*, 2011**a*,b*) and *N*-(aryl)- arylsulfonamides (Gowda *et al.*, 2005), the crystal structure of *N*,*N*-bis(4-chlorophenylsulfonyl)-suberamide has been determined (I) (Fig. 1).

In the two C—SO_2_—NH—CO—CH_2_—CH_2_—CH_2_— central amide and aliphatic segments of the structure, all the N—H, C=O and C—H bonds in the amide and aliphatic segments are *anti* to the adjacent bonds, similar to that observed in *N*,*N*-bis(2-chlorophenylsulfonyl)-suberamide (II) (Rodrigues *et al.*, 2011*b*) and *N*,*N*-bis(2-chlorophenylsulfonyl)-adipamide (III) (Rodrigues *et al.*, 2011*a*). The orientations of sulfonamide groups with respect to the attached phenyl rings are given by the torsion angles of C2—C1—S1—N1 = -113.9 (4)° and C6—C1—S1—N1 = 67.2 (3)°. The molecule is bent at the S atom with the C1—S1—N1—C7 torsion angle of -80.6 (4)°, compared to the values of 68.2 (2)° in (II) and -65.1 (6)° in (III). In (I), the aliphatic chain is linear with the C7—C8—C9—C10 torsion angle of -179.4 (4)°.

The dihedral angle between the benzene ring and the SO_2_—NH—C(O)—CH_2_—CH_2_—CH_2_ segment in the two halves of the molecule is 79.5 (2)°, compared to the values of 77.5 (1)° in (II) and 89.6 (2)° in (III).

The structure shows simultaneous of N—H···O(C) and N—H···O(S) intermolecular hydrogen bonds (Table 1), which link the molecules into infinite chains along the *b*-axis.

### Experimental

*N*,*N*-Bis(4-chlorophenylsulfonyl)-suberamide was prepared by refluxing a mixture of suberic acid (octanedioic acid) (0.01 mol) with 4-chlorobenzenesulfonamide (0.02 mol) and POCl_3_ for 1 hr on a water bath. The reaction mixture was allowed to cool and  ether added to it. The solid product was filtered and washed thoroughly with ether and hot ethanol. The compound was recrystallized to the constant melting point and characterized by its infrared and NMR spectra.

Needle like colorless single crystals used in the X-ray diffraction studies were grown by a slow evaporation of a solution of the compound in ethanol at room temperature.

### Refinement

The H atom of the NH group was located in a difference map and later restrained to N—H = 0.86 (2) Å. The other H atoms were positioned with idealized geometry using a riding model with the aromatic C—H = 0.93Å and the methylene C—H = 0.97 Å. All H atoms were refined with isotropic displacement parameters (set to 1.2 times of the *U*_eq_ of the parent atom).

### Crystal data

**Table:**

C_20_H_22_Cl_2_N_2_O_6_S_2_	*F*(000) = 540
*M**_r_* = 521.42	*D*_x_ = 1.511 Mg m^−^^3^
Monoclinic, *P*2_1_/*c*	Mo *K*α radiation, λ = 0.71073 Å
Hall symbol: -P 2ybc	Cell parameters from 1226 reflections
*a* = 21.925 (4) Å	θ = 2.8–27.9°
*b* = 5.5855 (8) Å	µ = 0.51 mm^−^^1^
*c* = 9.381 (1) Å	*T* = 293 K
β = 93.91 (1)°	Needle, colourless
*V* = 1146.1 (3) Å^3^	0.48 × 0.14 × 0.06 mm
*Z* = 2	

### Data collection

**Table:**

Oxford Diffraction Xcalibur diffractometer with Sapphire CCD detector	2081 independent reflections
Radiation source: fine-focus sealed tube	1522 reflections with *I* > 2σ(*I*)
graphite	*R*_int_ = 0.029
Rotation method data acquisition using ω scans.	θ_max_ = 25.3°, θ_min_ = 2.8°
Absorption correction: multi-scan (*CrysAlis RED*; Oxford Diffraction, 2009)	*h* = −24→26
*T*_min_ = 0.793, *T*_max_ = 0.970	*k* = −6→5
3854 measured reflections	*l* = −11→9

### Refinement

**Table:**

Refinement on *F*^2^	Primary atom site location: structure-invariant direct methods
Least-squares matrix: full	Secondary atom site location: difference Fourier map
*R*[*F*^2^ > 2σ(*F*^2^)] = 0.063	Hydrogen site location: inferred from neighbouring sites
*wR*(*F*^2^) = 0.115	H atoms treated by a mixture of independent and constrained refinement
*S* = 1.18	*w* = 1/[σ^2^(*F*_o_^2^) + (0.*P*)^2^ + 2.6128*P*] where *P* = (*F*_o_^2^ + 2*F*_c_^2^)/3
2081 reflections	(Δ/σ)_max_ = 0.009
148 parameters	Δρ_max_ = 0.29 e Å^−^^3^
1 restraint	Δρ_min_ = −0.32 e Å^−^^3^

### Special details

**Table:**

Geometry. All e.s.d.'s (except the e.s.d. in the dihedral angle between two l.s. planes) are estimated using the full covariance matrix. The cell e.s.d.'s are taken into account individually in the estimation of e.s.d.'s in distances, angles and torsion angles; correlations between e.s.d.'s in cell parameters are only used when they are defined by crystal symmetry. An approximate (isotropic) treatment of cell e.s.d.'s is used for estimating e.s.d.'s involving l.s. planes.
Refinement. Refinement of *F*^2^ against ALL reflections. The weighted *R*-factor *wR* and goodness of fit *S* are based on *F*^2^, conventional *R*-factors *R* are based on *F*, with *F* set to zero for negative *F*^2^. The threshold expression of *F*^2^ > σ(*F*^2^) is used only for calculating *R*-factors(gt) *etc*. and is not relevant to the choice of reflections for refinement. *R*-factors based on *F*^2^ are statistically about twice as large as those based on *F*, and *R*- factors based on ALL data will be even larger.

### Fractional atomic coordinates and isotropic or equivalent isotropic displacement parameters (Å^2^)

**Table:**

	*x*	*y*	*z*	*U*_iso_*/*U*_eq_	
Cl1	0.04045 (6)	0.7957 (3)	0.3395 (2)	0.1014 (6)	
S1	0.26694 (5)	0.1182 (2)	0.39060 (10)	0.0397 (3)	
O1	0.25535 (13)	−0.0692 (5)	0.4882 (3)	0.0482 (8)	
O2	0.28271 (13)	0.0610 (5)	0.2497 (3)	0.0486 (8)	
O3	0.35809 (14)	0.4648 (6)	0.2761 (3)	0.0608 (10)	
N1	0.32305 (15)	0.2771 (6)	0.4690 (3)	0.0397 (8)	
H1N	0.3190 (18)	0.284 (7)	0.559 (2)	0.048*	
C1	0.20287 (18)	0.3113 (8)	0.3787 (4)	0.0405 (10)	
C2	0.1531 (2)	0.2614 (9)	0.4551 (5)	0.0573 (13)	
H2	0.1534	0.1285	0.5148	0.069*	
C3	0.1026 (2)	0.4101 (10)	0.4425 (6)	0.0694 (15)	
H3	0.0686	0.3776	0.4932	0.083*	
C4	0.1034 (2)	0.6059 (9)	0.3547 (6)	0.0596 (13)	
C5	0.1531 (2)	0.6559 (9)	0.2780 (5)	0.0551 (12)	
H5	0.1527	0.7886	0.2181	0.066*	
C6	0.2034 (2)	0.5079 (8)	0.2906 (4)	0.0470 (11)	
H6	0.2374	0.5407	0.2399	0.056*	
C7	0.35867 (17)	0.4445 (8)	0.4041 (4)	0.0378 (10)	
C8	0.39757 (17)	0.5930 (8)	0.5078 (4)	0.0389 (10)	
H8A	0.4223	0.4876	0.5703	0.047*	
H8B	0.3713	0.6846	0.5665	0.047*	
C9	0.43924 (18)	0.7634 (8)	0.4345 (4)	0.0409 (10)	
H9A	0.4651	0.6715	0.3749	0.049*	
H9B	0.4144	0.8693	0.3726	0.049*	
C10	0.47944 (17)	0.9135 (8)	0.5373 (4)	0.0394 (10)	
H10A	0.5048	0.8079	0.5984	0.047*	
H10B	0.4537	1.0041	0.5978	0.047*	

### Atomic displacement parameters (Å^2^)

**Table:**

	*U*^11^	*U*^22^	*U*^33^	*U*^12^	*U*^13^	*U*^23^
Cl1	0.0516 (8)	0.0761 (11)	0.1751 (18)	0.0094 (8)	−0.0036 (9)	−0.0021 (11)
S1	0.0432 (6)	0.0422 (6)	0.0343 (5)	−0.0088 (5)	0.0065 (4)	−0.0047 (5)
O1	0.0551 (18)	0.0452 (19)	0.0447 (16)	−0.0108 (15)	0.0069 (14)	0.0030 (14)
O2	0.0577 (19)	0.054 (2)	0.0353 (15)	−0.0070 (15)	0.0085 (13)	−0.0108 (14)
O3	0.063 (2)	0.086 (3)	0.0328 (16)	−0.0361 (19)	0.0026 (14)	0.0065 (16)
N1	0.0386 (18)	0.052 (2)	0.0298 (16)	−0.0161 (17)	0.0076 (15)	−0.0034 (17)
C1	0.042 (2)	0.043 (3)	0.036 (2)	−0.010 (2)	0.0002 (18)	−0.005 (2)
C2	0.044 (3)	0.064 (3)	0.065 (3)	−0.009 (3)	0.013 (2)	0.011 (3)
C3	0.041 (3)	0.078 (4)	0.091 (4)	−0.008 (3)	0.018 (3)	0.006 (3)
C4	0.040 (3)	0.054 (3)	0.083 (3)	−0.007 (2)	−0.009 (2)	−0.010 (3)
C5	0.063 (3)	0.044 (3)	0.057 (3)	−0.010 (2)	−0.004 (2)	0.004 (2)
C6	0.044 (3)	0.050 (3)	0.047 (2)	−0.008 (2)	0.007 (2)	−0.006 (2)
C7	0.028 (2)	0.050 (3)	0.036 (2)	−0.0030 (19)	0.0049 (17)	−0.0005 (19)
C8	0.035 (2)	0.049 (3)	0.034 (2)	−0.005 (2)	0.0056 (17)	−0.004 (2)
C9	0.040 (2)	0.044 (3)	0.038 (2)	−0.010 (2)	0.0075 (17)	−0.0012 (19)
C10	0.035 (2)	0.047 (3)	0.036 (2)	−0.005 (2)	0.0071 (17)	−0.0043 (19)

### Geometric parameters (Å, °)

**Table:**

Cl1—C4	1.738 (5)	C4—C5	1.375 (6)
S1—O1	1.425 (3)	C5—C6	1.377 (6)
S1—O2	1.425 (3)	C5—H5	0.9300
S1—N1	1.649 (3)	C6—H6	0.9300
S1—C1	1.769 (4)	C7—C8	1.499 (5)
O3—C7	1.205 (4)	C8—C9	1.516 (5)
N1—C7	1.386 (5)	C8—H8A	0.9700
N1—H1N	0.852 (18)	C8—H8B	0.9700
C1—C2	1.374 (5)	C9—C10	1.515 (5)
C1—C6	1.375 (6)	C9—H9A	0.9700
C2—C3	1.382 (7)	C9—H9B	0.9700
C2—H2	0.9300	C10—C10^i^	1.525 (7)
C3—C4	1.370 (7)	C10—H10A	0.9700
C3—H3	0.9300	C10—H10B	0.9700
			
O1—S1—O2	119.75 (18)	C5—C6—C1	119.4 (4)
O1—S1—N1	105.61 (17)	C5—C6—H6	120.3
O2—S1—N1	108.29 (17)	C1—C6—H6	120.3
O1—S1—C1	108.25 (18)	O3—C7—N1	122.2 (4)
O2—S1—C1	108.65 (18)	O3—C7—C8	124.1 (4)
N1—S1—C1	105.37 (19)	N1—C7—C8	113.6 (3)
C7—N1—S1	126.4 (3)	C7—C8—C9	112.8 (3)
C7—N1—H1N	120 (3)	C7—C8—H8A	109.0
S1—N1—H1N	110 (3)	C9—C8—H8A	109.0
C2—C1—C6	121.0 (4)	C7—C8—H8B	109.0
C2—C1—S1	119.9 (4)	C9—C8—H8B	109.0
C6—C1—S1	119.1 (3)	H8A—C8—H8B	107.8
C1—C2—C3	119.6 (5)	C10—C9—C8	113.6 (3)
C1—C2—H2	120.2	C10—C9—H9A	108.8
C3—C2—H2	120.2	C8—C9—H9A	108.8
C4—C3—C2	119.3 (4)	C10—C9—H9B	108.8
C4—C3—H3	120.4	C8—C9—H9B	108.8
C2—C3—H3	120.4	H9A—C9—H9B	107.7
C3—C4—C5	121.2 (5)	C9—C10—C10^i^	113.2 (4)
C3—C4—Cl1	119.7 (4)	C9—C10—H10A	108.9
C5—C4—Cl1	119.1 (4)	C10^i^—C10—H10A	108.9
C6—C5—C4	119.5 (4)	C9—C10—H10B	108.9
C6—C5—H5	120.3	C10^i^—C10—H10B	108.9
C4—C5—H5	120.3	H10A—C10—H10B	107.7
			
O1—S1—N1—C7	165.0 (3)	C2—C3—C4—Cl1	−179.4 (4)
O2—S1—N1—C7	35.6 (4)	C3—C4—C5—C6	−0.7 (7)
C1—S1—N1—C7	−80.6 (4)	Cl1—C4—C5—C6	179.3 (3)
O1—S1—C1—C2	−1.3 (4)	C4—C5—C6—C1	0.6 (7)
O2—S1—C1—C2	130.2 (3)	C2—C1—C6—C5	−0.5 (6)
N1—S1—C1—C2	−113.9 (4)	S1—C1—C6—C5	178.4 (3)
O1—S1—C1—C6	179.8 (3)	S1—N1—C7—O3	−11.8 (6)
O2—S1—C1—C6	−48.7 (4)	S1—N1—C7—C8	168.7 (3)
N1—S1—C1—C6	67.1 (3)	O3—C7—C8—C9	−3.3 (6)
C6—C1—C2—C3	0.5 (7)	N1—C7—C8—C9	176.2 (3)
S1—C1—C2—C3	−178.5 (4)	C7—C8—C9—C10	−179.4 (3)
C1—C2—C3—C4	−0.5 (8)	C8—C9—C10—C10^i^	−179.2 (4)
C2—C3—C4—C5	0.6 (8)		

Symmetry codes: (i) −*x*+1, −*y*+2, −*z*+1.

### Hydrogen-bond geometry (Å, °)

**Table:**

*D*—H···*A*	*D*—H	H···*A*	*D*···*A*	*D*—H···*A*
N1—H1N···O2^ii^	0.85 (2)	2.19 (3)	2.975 (4)	153 (4)
N1—H1N···O3^ii^	0.85 (2)	2.57 (3)	3.227 (4)	135 (3)

Symmetry codes: (ii) *x*, −*y*+1/2, *z*+1/2.
